# Supplementary material for: Preterm Infants on Early Solid Foods and Neurodevelopmental Outcome—A Secondary Outcome Analysis of a Randomized Controlled Trial
Source: Nutrients. 2024 May 19;16(10):1528. doi: 10.3390/nu16101528 (PMC11124080; doi:10.3390/nu16101528)
Supplement: Supplementary file 1 [file nutrients-16-01528-s001.zip › nutrients-3008441-supplementary.pdf]

Table S1. Results of the linear mixed-effects models (fixed and random intercepts) for the Bayley-III scales at 1 and 2 years corrected and 2 years 4 months uncorrected age

	**1 year corrected age**			**2 years corrected age**			**3 years 4 months uncorrected age**		
	**Motor**	**Cognitive**	**Language**	**Motor**	**Cognitive**	**Language**	**Motor**	**Cognitive**	**Language**
Intercept	103 (20)	96 (23)	81 (21)	70 (21)	19 (27)	8 (33)	20 (26)	78 (25)	14 (29)
Group (early group)	-0.01 (2.91)	-0.79 (3.27)	-1.84 (3.00)	1.29 (3.00)	-0.59 (3.82)	-2.33 (4.57)	0.09 (3.40)	-2.26 (3.21)	-4.85 (3.72)
Nutrition at discharge (formula)	1.25 (3.61)	0.36 (4.07)	-3.60 (3.73)	-2.71 (3.79)	-9.71 (4.81)	-4.60 (5.82)	-0.94 (4.36)	-5.69 (4.14)	-7.58 (4.82)
Nutrition at discharge (mixed)	1.23 (3.51)	-0.70 (3.92)	-6.40 (3.60)	-1.44 (3.74)	-4.80 (4.73)	-7.58 (5.67)	-0.31 (3.98)	-1.71 (3.79)	-5.03 (4.30)
Gestational age (days)	-0.04 (0.10)	0.01 (0.12)	0.10 (0.11)	0.11 (0.11)	0.35 (0.14)	0.29 (0.17)	0.27 (0.13)	0.03 (0.12)	0.29 (0.14)
Sex (male)	0.69 (2.65)	-0.72 (2.71)	-2.87 (2.83)	0.02 (2.92)	-5.10 (3.41)	-2.69 (3.77)	0.09 (3.35)	0.64 (3.00)	2.84 (3.39)
Highest educational level of both parents (middle school)	-0.47 (5.31)	-3.58 (5.93)	1.34 (5.64)	1.73 (6.05)	7.30 (8.13)	18.71 (9.58)	9.05 (6.88)	9.87 (6.58)	14.34 (7.59)
Highest educational level of both parents (secondary school)	0.23 (5.92)	-2.24 (6.61)	-2.85 (6.22)	-0.62 (6.50)	13.68 (8.83)	27.09 (10.13)	15.50 (7.43)	13.41 (7.13)	23.47 (8.32)
Highest educational level of both parents (higher than secondary school)	-5.58 (5.13)	-6.09 (5.72)	0.05 (5.45)	-0.72 (5.80)	12.79 (7.78)	25.76 (9.13)	9.16 (6.70)	9.81 (6.43)	18.74 (7.31)

	1 year corrected age			2 years corrected age			3 years 4 months uncorrected age		
	**Motor**	**Cognitive**	**Language**	**Motor**	**Cognitive**	**Language**	**Motor**	**Cognitive**	**Language**
High grade IVH	-29 (5)	-23 (5)	-15 (6)	-25 (6)	-15 (7)	-6 (7)	-18 (7)	-22 (6)	-17 (7)
Random Intercept for siblings of multiple births, SD	12.14	15.29	10.69	8.68	15.70	20.11	8.62	10.85	12.96
N (total)	144	143	143	129	136	124	102	108	104

Results of the linear mixed-effects model (fixed and random intercepts), standard error (SE) in brackets, Group: early vs late group, Nutrition at discharge: mother's own milk vs formula vs mixed, Sex: male vs female, Highest educational level of both parents: none vs middle school vs secondary school vs higher than secondary school.

Table S2. Bayley-III scales of infant development at 1 and 2 years corrected and 3 years 4 months uncorrected age according to nutrition at discharge.

Parameter		Early group		Late group	
		Breastfeeding n = 24	Formula n = 48	Breastfeeding n = 25	Formula n = 44
**1 year** corrected age	Cognitive	90 (76-99)	90 (80-105)	90 (75-100)	92.5 (81-105)
	Language	100 (87-106)	97 (81-103)	94 (78-102)	97 (85-103)
	Motor	96 (82-103)	98 (85-103)	92 (75-105)	96 (86-106)
		n = 21	n = 44	n = 24	n = 44
**2 years** corrected age	Cognitive	90 (73-100)	85 (75-100)	85 (71-110)	80 (68-100)
	Language	78 (53-100)	81 (62-92)	81 (63-92)	75 (60-97)
	Motor	85 (81-98)	89 (82-100)	85 (76-103)	89 (76-106)
		n = 19	n = 31	n = 22	n = 38
**3 years 4 months** uncorrected age	Cognitive	90 (85-100)	95 (81-100)	95 (80-100)	90 (75-100)
	Language	84 (75-94)	81 (71-97)	87 (75-94)	84 (69-94)
	Motor	79 (70-87)	82 (67-96)	82 (70-89)	82 (70-89)

Data are presented as median with the 25. and the 75. percentile in parenthesis. * p-values <.05

Table S3. Bayley-III scales of infant development at 1 and 2 years corrected and 3 years 4 months uncorrected age according to sex.

Parameter		Early group		Late group	
		Female<br>n = 31	Male<br>n = 47	Female<br>n = 35	Male<br>n = 39
**1 year** corrected age	Cognitive	93 (85-104)	90 (78-103)	95 (80-100)	90 (73-106)
	Language	97 (88-103)	97 (75-105)	97 (84-103)	97 (80-103)
	Motor	94 (86-103)	102 (81-104)	100 (85-103)	92 (83-106)
		n = 31	n = 41	n = 35	n = 37
**2 years** corrected age	Cognitive	85 (75-101)	90 (75-100)	90 (75-110)*	75 (55-99)*
	Language	78 (66-91)	81 (52-99)	84 (65-99)*	66 (48-91)*
	Motor	85 (84-100)	92 (82-103)	91 (85-103)	82 (69-106)
		n = 20	n = 31	n = 31	n = 33
**3 years 4 months** uncorrected age	Cognitive	90 (85-100)	95 (81-104)	95 (80-100)	90 (75-100)
	Language	84 (69-94)	84 (75-97)	84 (75-91)	87 (69-99)
	Motor	81 (69-94)	82 (70-92)	82 (73-89)	85 (67-89)

Data are presented as median with the 25. and the 75. percentile in parenthesis. * p-value <.05

Table S4. Baseline characteristics & neonatal morbidity of infants with and without follow up data at 3 years 4 months of age.

**Parameter**	**Follow Up** (n=116)	**No Follow Up** (n=40)
Obstetric and parental parameters		
Multiple pregnancy	41 (35.3)	14 (35)
Cesarean delivery	111 (95.7)	32 (80)*
Prenatal steroids (full course)	67 (57.8)	24 (60)
Premature rupture of membranes	49 (42.2)	17 (42.5)
Preeclampsia	9 (7.8)	6 (15)
Age of mother at birth	33 [± 6]	33 [± 5]
Age of father at birth	36 [± 7]	37 [± 6]
Education mother		
No graduation/school diploma	14 (12.1)	7 (17.5)
Middle school	34 (29.3)	17 (42.5)
Secondary school	23 (19.8)	5 (12.5)
Post-secondary school	41 (35.3)	10 (25)
Education father		
No graduation/school diploma	8 (6.9)	6 (15)
Middle school	50 (43.1)	17 (42.5)
Secondary school	17 (14.7)	3 (7.5)
Post-secondary school	34 (29.3)	12 (30)
Neonatal parameters		
Male sex	65 (56)	23 (57.5)
Gestational age (days)	188 [± 14] – 26+6	198 [± 15] – 28+2*
Birth weight (g)	904 [± 243]	1047 [± 257]*
Small for gestational age	7 (6)	5 (12.5)
Gestational age (days) at discharge	265 [± 15]	254 [± 21]*
Breast milk feeding at discharge	41 (35.3)	8 (20)
Neonatal morbidity		
NEC grade I & II	2 (1.7)	2 (5)
PDA	46 (39.7)	9 (22.5)
ROP ≥ grade III	7 (6)	1 (2.5)
IVH ≥ grade II	15 (12.9)	0 (0)
PVL	2 (1.7)	0 (0)

Categorical data are presented as numbers with percentages in round parentheses. Continuous data are presented as the mean ± standard deviation in squared parentheses. * p-value <.05

IVH – intraventricular hemorrhage, NEC – necrotizing enterocolitis, PDA – persisting ductus arteriosus, PVL – periventricular leukomalacia, ROP – retinopathy of prematurity, SGA – small for gestational age (weight at birth <10th percentile)
